# Association of the *IL1A* and *IL6* polymorphisms with posttraining changes in body mass, composition, and biochemical parameters in Caucasian women

**DOI:** 10.5114/biolsport.2024.131415

**Published:** 2023-09-21

**Authors:** Agata Leońska-Duniec, Weronika Lepionka, Andrzej Brodkiewicz, Maciej Buryta

**Affiliations:** 1 Faculty of Physical Education, Gdansk University of Physical Education and Sport, 80-336 Gdansk, Poland; 2 Department of Pediatrics, Child Nephrology, Dialisotherapy and Management of Acute Poisoning, Pomeranian Medical University, 70-204 Szczecin, Poland; 3 Institute of Physical Culture Sciences, University of Szczecin, 70-453 Szczecin, Poland

**Keywords:** Sports genetics, Interleukins, Polymorphisms, Physical activity, Obesity related traits

## Abstract

Polymorphisms located in IL1A and IL6 are promising markers of obesity-related traits; however, studies concerning their potential impact on the effectiveness of lifestyle interventions are lacking. Therefore, the aim was to examine the association between the polymorphic sites located in IL1A (rs1800587) and IL6 (rs1800795, rs1800796, and rs1800797) and the body’s response to a 12-week training program. We studied the genotype distribution in a group of 168 Caucasian females in whom body mass and composition parameters, the lipid profile, and glucose levels were measured before and after the exercise period. Our results showed that carriers of the IL1A rs1800597 CC genotype exhibited a significant decrease in total body water (TBW) in response to training (p = 0.045). Additionally, carriers of the IL6 rs1800797 GG and GA genotypes demonstrated a posttraining decrease in body mass index (BMI) (p = 0.039). Haplotype analysis revealed that only rare haplotypes, namely, GGA, CGG and CCG (rs1800795, rs1800796, and rs1800797, respectively), were linked to changes in phenotype, yet assessing individual haplotype effects was not possible. Studies of the interactions between these genes showed that carrying the TC-GG genotype (rs1800587-rs1800795 and rs1800587-rs1800796) may be associated with greater posttraining decreases in fat mass percentage (%FM) and fat-free mass (FM). Carriers of the CC-CG genotype (rs1800587-rs1800795) had significantly greater changes in triglycerides (TGL) over the training period. Our study showed that the IL1A and IL6 genotypes, either individually, in haplotype, or in gene-gene combination, may modify training-induced changes in body mass, composition, glucose levels, and the lipid profile in healthy women.

## INTRODUCTION

Cytokines have been defined as “one group of protein cell regulators variously called lymphokines, monokines, interleukins, interferons and chemokines produced by a wide variety of cells in the body that play an important role in many physiologic responses” [1]. They consist of over 200 secreted factors that create a dynamic highly complex and intricate network of elements controlling the immune response [1, 2]. Circulating levels of cytokines reach a characteristic profile in specific metabolic states, e.g., in obesity, which is considered a chronic, low-grade inflammatory disease [3]. Additionally, it has been demonstrated that physical activity and weight change induce considerable variations in the levels of several cytokines [4]. Physiologically and anatomically, fat and muscle tissues are connected and play an important role in metabolism [5]. Taken together, these proteins and the genes encoding them are very promising subjects of research on posttraining changes in body mass, composition, and biochemical parameters.

Interleukin-1 (IL-1) is a superfamily of pleiotropic cytokines with numerous functions in mostly immunity, inflammation, and hemopoiesis. This family contains 11 members, including interleukin-1 alpha (IL-1α), which is a major proinflammatory cytokine [6]. This interleukin is produced in cells including macrophages, monocytes, neutrophils, and hepatocytes. It has been shown to contribute to obesity and insulin resistance. Elevated blood serum IL-1α levels were associated with increased body mass in humans [3, 7]. The *IL1A* gene, similar to other IL-1 family genes, is located on chromosome 2 (2q14.1) and contains 8 exons. Numerous studies have described associations between single nucleotide polymorphisms (SNPs) of *IL1A* and numerous diseases, such as obesity [8, 9], metabolic syndrome [10], cardiovascular disorders [11], asthma [12], and cancer [13]. One of the functional polymorphisms affecting *IL1A* expression is rs1800587 (-889 C > T), which is localized in the promoter region of the gene [14].

Interleukin-6 (IL-6) is a multitarget, pleiotropic cytokine that is a key player in the control of immune responses, inflammation, hematopoiesis, and host defense mechanisms. It is secreted by white adipose tissue, skeletal muscle, and the liver [15]. Elevated IL-6 levels have been correlated with increased body mass, waist circumference, and free fatty acid levels [16], with a decrease in plasma circulating IL-6 after weight loss [17]. The protein is encoded by the *IL6* gene, which is localized on the short arm of chromosome 7 (7p15.3) and contains 5 exons. The promoter region includes numerous SNPs that affect gene transcription in specific types of cells [18]. For the purposes of this article, three polymorphisms, namely, rs1800795 (-174 G > C), rs1800796 (-572 G > C), and rs1800797 (-597 A > G), with the greatest impact on *IL6* gene expression, were selected.

Although the *IL1A* and *IL6* polymorphisms are promising genetic markers of obesity-related traits, data are lacking concerning the potential impact of these SNPs on the effectiveness of lifestyle interventions such as regular physical activity. Thus, the aim of this study was to establish whether the *IL1A* (rs1800587) and *IL6* (rs1800795, rs1800796, and rs1800797) polymorphisms would affect the posttraining changes in selected body mass, composition, and biochemical measurements. To examine the possible association between genotypes, haplotypes, and interactions between these two genes and physical outcomes, we evaluated the allele and genotype distribution in a group of young healthy females participating in 12 weeks of aerobic training.

## MATERIALS AND METHODS

### Ethics statement

The experiment was approved by The Ethics Committee of the Regional Medical Chamber in Szczecin (no. 09/KB/IV/2011 and 01/KB/VI/2017) and was conducted ethically according to the Strengthening the Reporting of Genetic Association studies statement (STREGA) and World Medical Association Declaration of Helsinki. All participants obtained an information sheet about the aim, procedures, risks and benefits of the experiment, and a written consent form. Pseudonymization was applied as the method of data protection.

### Participants

One hundred sixty eight Polish Caucasian females (n = 168; age: 21 ± 1 years; body height: 168 ± 2 cm; body mass: 61 ± 2 kg) were chosen for this study. The following inclusion criteria were considered: low level of physical activity self-reported with the use of the Global Physical Activity Questionnaire (according to the World Health Organization in the Polish adaptation); no metabolic, musculoskeletal or neuromuscular diseases; refrained from using medications and supplements for 6 months prior to the start of the experiment; as well as nonsmokers.

### Dietary program

The participants who met the inclusion criteria took part in a dietary program and were expected to keep a balanced diet based on their personal dietary plan which was established during a nutritional meeting involving a recommendation of an adequate diet matched with personal energy needs and nutritional status. The medium daily macronutrient ratio was proposed (expressed as a percentage of total calories): 45–65% from carbohydrates, 20–35% from fat (reducing the intake of saturated fats and increasing the intake of unsaturated fats), and 10–20% from protein. The women were also instructed to maintain a daily cholesterol intake of less than 300 mg with a minimum dietary fiber intake of 25 g. The quality and quantity of food and drink were assessed during weekly consultations.

### Training Phase

At first, a week-long familiarization period (3 training units, 30 min each, at ~50% of maximum heart rate – HRmax) preceded a proper training. A 12-week (36 training units) experimental training program was divided as follows:

– stage 1: 1–3 weeks (9 training units), 60 min each, at 50–60% of HRmax, tempo 135–140 beats per minute (BPM);
– stage 2: 4–6 weeks (9 training units), 60 min each, at 60–70% of HRmax, tempo 140–152 BPM;
– stage 3: 7–9 weeks (9 training units), 60 min each, at 65–75% of HRmax, tempo 145–158 BPM;
– stage 4: 10–12 weeks (9 training units), 60 min each, at 65–80% of HRmax, tempo 145–160 BPM.

Each training unit included a warm-up (10 min), the main aerobic exercise (43 min), and cool-down period (7 min). A program combining high and low impact styles was described in detail previously [19].

### Body mass and composition measurements

Before and after the realization of the 12-week training program, the chosen body mass and composition parameters were assessed with the bioimpedance method using an electronic scale Tanita TBF 300 M (Arlington Heights, Illinois, United States) as previously described [19]. The following parameters were noted:

– total body mass (BM, kg);
– body mass index (BMI, -);
– basal metabolic rate (BMR, kcal);
– fat mass (FM, kg);
– fat free mass (FFM, kg);
– fat mass percentage (%FM, %);
– total body water (TBW, kg).

### Biochemical Analyses

Biochemical analyses were performed before the beginning of the training program and repeated after the 36th training unit. Fasting blood samples were obtained from the elbow vein in the morning.

The analyses were performed at once after the blood collection as previously described in detail [19]. The selected parameters received using the Random Access Automatic Biochemical Analyzer for Clinical Chemistry and Turbidimetry A15 (BioSystems S.A., Barcelona, Spain) were:

– total cholesterol (TC, mg/dL);
– triglycerides (TGL, mg/dL);
– high-density lipoprotein (HDL, mg/dL);
– low-density lipoprotein (LDL, mg/dL);
– blood glucose (BG, mg/dL).

### Genetic Analyses

Genomic DNA was extracted from the buccal cells using a GenElute Mammalian Genomic DNA Miniprep Kit (Sigma, Steinheim, Germany) according to the manufacturer’s recommendation. All samples were genotyped in duplicate, using TaqMan® Pre-Designed SNP Genotyping Assays (Applied Biosystems, Waltham, MA, USA) on a C1000 Touch Thermal Cycler (Bio-Rad, Feldkirchen, Germany) instrument according to the manufacturer’s procedures. The assays (C___9546481_30, C___1839697_20, C___9546481_30, and C___1839695_20) included primers and fluorescently labeled (FAM and VIC) probes to discriminate the *IL1A* and *IL6* alleles.

### Statistical analyses

Statistical analyses were performed in R (https://cran-r.project.org, accessed on 7 October 2021, version 4.1.0). An HWChisq function from Hardy-Weinberg v. 1.7.4 R package was used to test for Hardy– Weinberg equilibrium. No variants violating HW equilibrium were observed. To check the normality of the distribution of the random variable, the Shapiro-Wilk test was used. The influence of the *IL1A* and *IL6* polymorphisms on training response was performed using two-way ANOVA with repeated measures with one between-subject factor (genotype) and one within-subject factor (time: before training vs after training) and FDR adjustment after. To test the significance of the effect of the applied training on the study variables, the Wilcoxon paired rank-order test was used. Haplotype analysis was conducted with haplo.stats v. 1.8.7 R package and haplo.glm regression function. Haplotypes for which the occurrence rate exceeded 1% were included. Percentage change overtraining was used as the dependent variable, while the *IL6* haplotypes were used as the independent variables. Interactions between genes were analyzed in a general linear model (GLM) using the following penetrance models: dominant model (DOM), recessive model (REC) and homozygote-heterozygote model (or heterozygote-homozygote, HOM-HET/HET-HOM). In addition, each of the four possible genotype combinations for the HOM-HET model was analyzed individually (HOM1-HET, HOM2-HET, HET-HOM1, HET-HOM2), where HOM1 is the homozygote for the risk allele in question and HOM2 is the homozygote for the major allele. The level of statistical significance was set at p < 0.05.

## RESULTS

### Individual SNPs analysis

Genotype and allele frequencies of the interleukin genes polymorphism are shown in Table 1–4. All markers variants were tested for Hardy-Weinberg equilibrium (HWE) and no significant deviations from theoretical frequencies were found (*IL1A* rs1800587 p = 0.852; *IL6* rs1800795 p = 0.438; rs1800796 p = 1.0; rs1800797 p = 0.438).

**TABLE 1:** Training responses by the *IL1A* rs1800587 genotypes.

Para- meter	TT (n = 85)		TC (n = 70)		CC (n = 13)		Genotype	Training	Genotype × training

	Pre	Post	Pre	Post	Pre	Post			
BM (kg)	60.3 ± 8.0	59.5 ± 7.9	61.4 ± 7.0	60.8 ± 6.9	58.1 ± 8.5	57.4 ± 8.3	0.289	< **0.0001**	0.864
BMI (-)	21.6 ± 2.4	21.4 ± 2.3	21.8 ± 2.4	21.6 ± 2.4	20.1 ± 2.4	19.9 ± 2.5	0.053	**0.0001**	0.926
%FM (%)	23.9 ± 5.5	22.2 ± 5.8	24.1 ± 5.3	23.1 ± 5.5	22.8 ± 5.9	21.2 ± 5.1	0.583 <	**0.0001**	0.120
FM (kg)	14.7 ± 5.2	13.6 ± 5.3	15.1 ± 4.9	14.4 ± 5.0	13.6 ± 5.3	12.6 ± 4.8	0.482	< **0.0001**	0.273
FFM (kg)	45.4 ± 3.4	46.0 ± 3.5	46.2 ± 2.9	46.6 ± 2.9	44.3 ± 3.2	44.6 ± 3.3	0.095	**0.005**	0.483
TBW (kg)	33.2 ± 2.7	33.7 ± 2.6	33.9 ± 2.1	34.1 ± 2.1	33.2 ± 3.8	32.8 ± 2.6	0.233	0.433	**0.045**
TC (mg/dL)	169 ± 26	169 ± 29	169 ± 24	167 ± 26	184 ± 19	174 ± 21	0.293	0.065	0.238
TGL (mg/dL)	76 ± 24	80 ± 27	83 ± 40	85 ± 33	90 ± 33	101 ± 72	0.094	0.130	0.696
HDL (mg/dL)	64 ± 13	61 ± 13	66 ± 14	62 ± 15	67 ± 12	60 ± 12	0.785	**0.0001**	0.737
LDL (mg/dL)	89 ± 22	92 ± 26	86 ± 22	88 ± 22	100 ± 23	93 ± 15	0.275	0.795	0.282
BG (mg/dL)	79 ± 10	76 ± 11	78 ± 10	76 ± 9	78 ± 13	72 ± 8	0.705	**0.001**	0.388

Mean ± standard deviation; p values (ANOVA) for main effects (genotype and training) and genotype × training interaction; bold p values –statistically significant differences (p < 0.05).

**TABLE 2:** Training responses by the *IL6* rs1800795 genotypes.

Para- meter	GG (n = 55)		CG (n = 78)		CC (n = 35)		Genotype	Training	Genotype × training

	Pre	Post	Pre	Post	Pre	Post			
BM (kg)	60.3 ± 8.4	59.5 ± 8.2	61.3 ± 8.0	60.5 ± 8.0	59.4 ± 5.0	58.9 ± 5.3	0.505	**< 0.0001**	0.488
BMI (-)	21.6 ± 3.0	21.3 ± 2.9	21.8 ± 2.2	21.6 ± 2.1	21.1 ± 1.9	21.0 ± 2.0	0.390	**< 0.0001**	0.109
%FM (%)	23.7 ± 6.1	22.5 ± 6.4	24.2 ± 5.4	22.7 ± 5.4	23.5 ± 4.5	22.2 ± 5.0	0.847	**< 0.0001**	0.831
FM (kg)	14.7 ± 5.8	13.9 ± 5.8	15.2 ± 5.1	14.1 ± 5.1	14.1 ± 3.6	13.2 ± 4.0	0.599	**< 0.0001**	0.562
FFM (kg)	45.6 ± 3.3	45.9 ± 3.2	46.0 ± 3.5	46.5 ± 3.5	45.0 ± 2.3	45.8 ± 2.8	0.383	**< 0.0001**	0.191
TBW (kg)	33.4 ± 2.4	33.7 ± 2.5	33.7 ± 2.5	34.0 ± 2.5	33.0 ± 2.9	33.5 ± 2.0	0.447	**0.002**	0.669
TC (mg/dL)	168 ± 21	166 ± 21	169 ± 27	168 ± 32	174 ± 25	173 ± 25	0.497	0.375	0.974
TGL (mg/dL)	83.1 ± 39.0	81.8 ± 27.7	77.2 ± 30.2	84.0 ± 42.9	82.5 ± 23.8	85.9 ± 24.2	0.824	0.270	0.376
HDL (mg/dL)	65.9 ± 12.3	61.9 ± 13.1	64.7 ± 13.6	59.7 ± 13.7	64.7 ± 14.7	63.1 ± 13.9	0.679	**0.00009**	0.306
LDL (mg/dL)	85.7 ± 18.8	88.0 ± 17.2	89.2 ± 23.5	91.5 ± 26.5	92.5 ± 23.1	92.4 ± 25.3	0.411	0.364	0.814
BG (mg/dL)	79.4 ± 10.9	77.2 ± 9.9	78.7 ± 9.6	75.8 ± 10.4	75.0 ± 8.0	72.3 ± 9.1	**0.034**	**0.002**	0.933

Mean ± standard deviation; p values (ANOVA) for main effects (genotype and training) and genotype × training interaction; bold p values – statistically significant differences (p < 0.05).

**TABLE 3:** Training responses by the *IL6* rs1800796 genotypes.

Para- meter	GG (n = 143)		CC+CG (n = 25)		Genotype	Training	Genotype × training

	Pre	Post	Prew	Post			
BM (kg)	60.3 ± 7.1	59.5 ± 7.0	62.3 ± 10.1	61.7 ± 9.7	0.207	**0.00009**	0.675
BMI (-)	21.5 ± 2.3	21.3 ± 2.3	22.1 ± 3.0	21.9 ± 2.9	0.211	**0.0004**	0.476
%FM (%)	23.8 ± 5.3	22.5 ± 5.4	24.1 ± 6.6	22.8 ± 6.9	0.794	**< 0.0001**	0.993
FM (kg)	14.7 ± 4.7	13.7 ± 4.8	15.6 ± 6.7	14.8 ± 6.7	0.349	**< 0.0001**	0.718
FFM (kg)	45.5 ± 3.0	45.9 ± 3.1	46.7 ± 4.0	47.1 ± 3.8	0.086	**0.001**	0.842
TBW (kg)	33.3 ± 2.5	33.7 ± 2.3	34.2 ± 2.9	34.6 ± 2.7	0.098	**0.017**	0.783
TC (mg/dL)	171 ± 24	169 ± 26	163 ± 26	165 ± 33	0.246	0.915	0.261
TGL (mg/dL)	79.8 ± 28.2	83.2 ± 34.3	82.6 ± 50	86.4 ± 38.9	0.643	0.317	0.955
HDL (mg/dL)	65.2 ± 13.4	61.4 ± 13.1	64.5 ± 13.2	59.3 ± 15.7	0.589	**0.0002**	0.535
LDL (mg/dL)	90.0 ± 22.3	90.8 ± 23.3	81.7 ± 18.9	88.9 ± 25.6	0.251	0.061	0.134
BG (mg/dL)	78.0±9.8	75.6 ± 10.2	78.8 ± 9.9	75.1 ± 9.3	0.929	**0.006**	0.561

Mean ± standard deviation; p values (ANOVA) for main effects (genotype and training) and genotype × training interaction; bold p values – statistically significant differences (p < 0.05).

**TABLE 4:** Training responses by the *IL6* rs1800797 genotypes.

Para- meter	GG (n = 55)		AG (n = 78)		AA (n = 35)		Genotype	Training	Genotype × training

	Pre	Post	Pre	Post	Pre	Post			
BM (kg)									
BM (kg)	60.8 ± 8.4	60.1 ± 8.1	61.0 ± 8.1	60.0 ± 8.0	59.5 ± 5.1	59.1 ± 5.4	0.717	**< 0.0001**	0.240
BMI (-)	21.6 ± 3.0	21.4 ± 2.9	21.8 ± 2.2	21.5 ± 2.1	21.0 ± 1.9	21.0 ± 2.0	0.397	**< 0.0001**	**0.039**
%FM (%)	24.0 ± 5.9	22.9 ± 6.1	24.0 ± 5.6	22.4 ± 5.5	23.4 ± 4.4	22.1 ± 5.0	0.813	**< 0.0001**	0.506
FM (kg)	15.0 ± 5.6	14.3 ± 5.6	15.0 ± 5.2	13.9 ± 5.2	14.0 ± 3.5	13.2 ± 4.0	0.586	**< 0.0001**	0.249
FFM (kg)	45.3.3 ±	46.0 ± 3.2	45.9 ± 3.4	46.3 ± 3.4	45.2 ± 2.4	46.0 ± 2.8	0.749	**< 0.0001**	0.191
TBW (kg)	33.5 ± 2.4	33.8 ± 2.5	33.6 ± 2.5	33.9 ± 2.5	33.1 ± 3.0	33.7 ± 2.1	0.780	**0.002**	0.635
TC (mg/dL)	169 ± 22	168 ± 26	170 ± 27	167 ± 30	172 ± 24	172 ± 24	0.774	0.516	0.680
TGL (mg/dL)	83.5 ± 38.9	83.1 ± 27.4	77.4 ± 30.2	83.6 ± 42.9	81.3 ± 24.1	84.8 ± 25.0	0.839	0.254	0.536
HDL (mg/dL)	66.0 ± 12.2	61.5 ± 13.0	64.6 ± 13.6	59.8 ± 13.7	64.8 ± 14.7	63.4 ± 13.9	0.680	**< 0.0001**	0.260
LDL (mg/dL)	86.1 ± 19.3	90.0 ± 19.9	89.9 ± 23.7	90.5 ± 25.6	90.5 ± 22.0	91.5 ± 24.7	0.769	0.253	0.627
BG (mg/dL)	79.2 ± 10.8	76.7 ± 10.1	79.0 ± 9.4	75.8 ± 9.9	74.7 ± 8.3	73.0 ± 10.3	0.061	**0.003**	0.780

Mean ± standard deviation; p values (ANOVA) for main effects (genotype and training) and genotype × training interaction; bold p values – statistically significant differences (p < 0.05).

To investigate the effect of training and polymorphisms of the *IL1A* and *IL6* genes a two-way ANOVA with repeated measures was conducted (for the *IL6* rs1800796 one rare homozygote and heterozygotes were pooled) (Tables 1–4). We found two significant (but not after FDR adjustment) genotype × training interactions suggesting a modulating effect of interleukin genes variants on training induced changes in the TBW (*IL1A* rs1800587; Table 1) and BMI (*IL6* rs1800797; Table 4). Carriers of the *IL1A* rs1800597 CC genotype exhibited a significant decrease in TBW in response to applied training in comparison to TC and TT genotypes (p = 0.045). The posttraining values of BMI were significantly lower compared with pre-training for the *IL6* rs1800797 GG and GA genotypes (p = 0.039).

### Haplotype analysis

Haplotypes were reconstructed for the *IL6* gene. Six haplotypes were present in 168 women. The three main haplotypes: GGG – 47.5%, CGA – 43.1%, GCG – 7.6% (rs1800795, rs1800796, rs1800797, respectively) with frequencies greater than 1% accounted for 98.2% of the total haplotypes. Examination of haplotype structure and frequencies across these *loci* suggested a high linkage between the rs1800795 and rs1800797, which was confirmed by a linkage disequilibrium (LD) pattern for the 1000G CEU population. Reconstructed haplotypes were tested for association with body composition- and lipid-related phenotypes. We did not find any significant associations for the main haplotypes. However, rare haplotypes comprising of haplotypes GGA, CGG and CCG were associated with smaller changes of several phenotypes under a recessive model (Table 5), yet estimating of individual haplotype effects was not possible.

**TABLE 5:** *IL6* haplotype analysis, a linear model with a relative change as the dependent variable, and baseline values (before training) as independent variables.

		CGA	GCG	Rare genotypes (GGA, CGG, CCG)
BM (kg)	OVER	0.17 (0.17), p = 0.339	0.36 (0.34), p = 0.290	0.48 (0.66), p = 0.472
	DOM	0.07 (0.26), p = 0.798	0.22 (0.35), p = 0.543	0.40 (0.66), p = 0.550
	REC	0.32 (0.29), p = 0.280	2.43 (1.52), p = 0.112	**-5.89 (1.84), p = 0.002**

BMI (-)	OVER	0.09 (0.06), p = 0.114	0.16 (0.11), p = 0.131	0.18 (0.21), p = 0.409
	DOM	0.04 (0.08), p = 0.659	0.11 (0.11), p = 0.328	0.14 (0.21), p = 0.532
	REC	0.17 (0.09), p = 0.059	0.77 (0.45), p = 0.093	**-1.99 (0.34), p < 0.0001**

%FM (%)	OVER	-0.11 (0.25), p = 0.656	0.02 (0.48), p = 0.962	0.70 (0.94), p = 0.456
	DOM	-0.30 (0.37), p = 0.430	-0.12 (0.50), p = 0.813	0.65 (0.94), p = 0.486
	REC	-0.03 (0.42), p = 0.953	2.61 (2.20), p = 0.236	**-7.51 (3.13), p = 0.017**

FM (kg)	OVER	-0.10 (0.18), p = 0.583	0.16 (0.35), p = 0.638	0.60 (0.68), p = 0.379
	DOM	-0.30 (0.27), p = 0.271	0.06 (0.36), p = 0.870	0.55 (0.86), p = 0.421
	REC	-0.01 (0.30), p = 0.961	2.06 (1.54), p = 0.184	**-6.63 (1.64), p < 0.0001**

FFM (kg)	OVER	0.26 (0.14), p = 0.062	0.24 (0.27), p = 0.362	-0.26 (0.53), p = 0.625
	DOM	0.32 (0.21), p = 0.135	0.22 (0.28), p = 0.434	-0.30 (0.53), p = 0.576
	REC	0.41 (0.23), p = 0.075	0.10 (1.19), p = 0.931	**5.49 (1.19), p < 0.0001**

TBW (kg)	OVER	0.14 (0.14), p = 0.346	0.29 (0.28), p = 0.300	0.14 (0.55), p = 0.794
	DOM	0.18 (0.22), p = 0.409	0.32 (0.29), p = 0.273	0.13 (0.55), p = 0.808
	REC	0.13 (0.25), p = 0.610	-0.40 (1.31), p = 0.763	0.17 (12.50), p = 0.989

TC (mg/dL)	OVER	1.20 (2.27), p = 0.597	2.15 (5.24), p = 0.682	12.37 (8.44), p = 0.145
	DOM	0.42 (3.40), p = 0.901	1.65 (5.48,) p = 0.763	1.84 (8.45), p = 0.163
	REC	2.16 (3.89), p = 0.579	1.59 (2.03), p = 0.938	**-7.11 (5.028e-03), p < 0.0001**

TGL (mg/dL)	OVER	2.14 (3.36), p = 0.524	0.02 (6.54), p = 0.997	-6.43 (12.70), p = 0.613
	DOM	4.52 (5.06), p = 0.373	2.00 (6.80), p = 0.769	-5.91 (12.69), p = 0.642
	REC	3.73 (4.54), p = 0.412	-32.92 (27.43), p = 0.232	**171 (17), p < 0.0001**

HDL (mg/dL)	OVER	0.76 (1.11), p = 0.498	-1.54 (2.15), p = 0.475	4.64 (4.22), p = 0.273
	DOM	-0.44 (1.68), p = 0.794	-2.12 (2.26), p = 0.349	4.03 (4.22), p = 0.341
	REC	2.99 (1.92), p = 0.121	-2.67 (1.00), p = 0.790	**-31.09 (5.504e-03), p < 0.0001**

LDL (mg/dL)	OVER	0.12 (2.07), p = 0.955	3.75 (4.06), p = 0.357	8.97 (9.37), p = 0.340
	DOM	-0.07 (3.13), p = 0.983	2.88 (4.40), p = 0.513	8.68 (9.82), p = 0.378
	REC	-0.76 (1.87), p = 0.833	18.59 (18.71), p = 0.322	**-6.54 (4.208e-03), p < 0.0001**

BG (mg/dL)	OVER	-1.16 (1.00), p = 0.247	-0.96 (2.13), p = 0.652	5.10 (3.87), p = 0.189
	DOM	-0.96 (1.49), p = 0.520	-0.57 (2.12), p = 0.796	5.32 (3.73), p = 0.156
	REC	-2.37 (1.72), p = 0.171	-1.48 (9.11), p = 0.871	**-20.73 (2.653e-03), p < 0.0001**

Table cells show model coefficients, coefficient’s standard error in parentheses and raw p values—statistically significant when p < 0.05. OVER- overdominant model; DOM- dominant model; REC – recessive model.

### Gene-gene interactions

Given a strong linkage disequilibrium between the rs1800795 and rs1800797 variants of the *IL6*, pairwise gene-gene interactions were examined across *IL1A* rs1800587, *IL6* rs1800795, and *IL6* rs1800796 variants. The *IL1A* rs1800587 had a significant interaction effect with *IL6* rs1800795 (Table 6) and with *IL6* rs1800796 (Table 7) on a change in %FM, FM, and TGL (only *IL1A* rs1800587 and *IL6* rs1800795) under the homozygote-heterozygote model. A change in %FM and FM over training period tended to be higher in carriers of the compound *IL1A* heterozygous (TC) and *IL6* homozygous (GG) genotype. Although these interactions were nominally significant none of them remained significant after multiple-test correction. In contrast, an effect of the *IL1A* rs1800587 and *IL6* rs1800795 interaction on a change in TGL remained significant after correction. Women carrying the *IL1A* risk allele homozygous CC genotype and *IL6* rs1800795 CG heterozygous genotype had significantly higher change in TGL over training period (coeff. 77.31, SE = 16.45, FDR p < 0.0001, Table 6).

**TABLE 6:** Analysis of the *IL1A* rs1800587 × *IL6* rs1800795 interaction.

	DOM	REC	HOM-HET	HOM1-HET CC-CG	HOM2-HET TT-CG	HET-HOM1 TC-CC	HET-HOM2 TC-GG
BM (kg)	0.15 (0.26), p = 0.577	0.59 (0.71), p = 0.407	0.06 (0.24), p = 0.807	-0.42 (0.92), p = 0.644	-0.12 (0.28), p = 0.677	0.40 (0.51), p = 0.433	0.16 (0.33), p = 0.626
	
BMI (-)	0.02 (0.08), p = 0.768	0.23 (0.23), p = 0.308	0.002 (0.08), p = 0.976	-0.16 (0.29), p = 0.592	-0.06 (0.09), p = 0.470	0.14 (0.16), p = 0.407	0.06 (0.11), p = 0.582
	
%FM (%)	0.23 (0.37), p = 0.537	-0.48 (1.01), p = 0.632	0.67 (0.34), p = 0.051	0.57 (1.30), p = 0.660	-0.19 (0.40), p = 0.636	1.18 (0.72), p = 0.103	**0.93 (0.46), p = 0.045**
	
FM (kg)	0.05 (0.27), p = 0.861	-0.10 (0.73), p = 0.891	0.41 (0.25), **p = 0.010**	0.21(0.94), p = 0.825	-0.16 (0.29), p = 0.570	0.66 (0.52), p = 0.213	**0.69 (0.33), p = 0.040**
	
FFM (kg)	-0.12 (0.21), p = 0.579	0.75 (0.57), p = 0.187	-0.13 (0.19), p = 0.497	-0.92 (0.73), p = 0.210	0.10 (0.22), p = 0.644	-0.56 (0.41), p = 0.168	-0.03 (0.26), p = 0.896
	
TBW (kg)	-0.25 (0.22), p = 0.246	-1.15 (0.58), p = 0.050	-0.19 (0.20), p = 0.348	-0.89 (0.75), p = 0.238	0.01 (0.23), p = 0.961	-0.49 (0.42), p = 0.244	-0.04 (0.27), p = 0.874
	
TC (mg/dL)	1.16 (3.38), p = 0.732	-10.46 (9.16), p = 0.255	-3.84 (3.09), p = 0.217	8.26 (11.72), p = 0.482	-2.99 (3.58), p = 0.404	-0.53 (6.61), p = 0.936	-3.79 (4.22), p = 0.370
	
TGL (mg/dL)	8.08 (5.01), p = 0.109	-8.32 (13.63), p = 0.542	1.26 (4.62), p = 0.786	**77.31 (16.45), p < 0.0001**	-3.71 (5.36), p = 0.491	5.34 (9.87), p = 0.589	-4.78 (6.31), p = 0.450
	
HDL (mg/dL)	-0.50 (1.68), p = 0.765	-3.11 (4.54), p = 0.493	0.46 (1.55), p = 0.767	-2.55 (5.82), p = 0.661	-1.27 (1.78), p = 0.477	5.24 (3.26), p = 0.109	0.79 (2.10), p = 0.706
	
LDL (mg/dL)	0.35 (3.12), p = 0.911	-4.16 (8.47), p = 0.624	-4.16 (2.85), p = 0.146	-3.49 (10.84), p = 0.748	-0.95 (3.31), p = 0.774	-5.46 (6.04), p = 0.368	-3.70 (3.91), p = 0.345
	
BG (mg/dL)	-0.37 (1.50), p = 0.803	-6.06 (4.00), p = 0.132	-0.68 (1.37), p = 0.619	-0.64 (5.17), p = 0.901	-0.82 (1.58), p = 0.607	-1.27 (2.92), p = 0.665	0.47 (1.86), p = 0.803

*IL1A* (rs1800587) – G risk allele, *IL6* (rs1800795) – C risk allele. Table cells show model coefficients, coefficient’s standard error in parentheses and raw p values—statistically significant when p < 0.05. DOM – dominant model: at least one risk allele at both *loci*; REC – recessive model: homozygotes for the risk allele at both *loci*; HOM-HET – homozygote-heterozygote model including four possible genotype combinations, where HOM1 is the homozygote for the risk allele and HOM2 is the homozygote for the major allele.

**TABLE 7:** Analysis of the *IL1A* rs1800587 × *IL6* rs1800796 interaction.

	DOM	REC	HOM-HET	HOM1-HET CC-CG	HET-HOM2 TC-GG	HET-HOM1 TC-CC	HET-HOM2 TC-GG
BM (kg)	0.33 (0.49), p = 0.497	Na	0.18 (0.24), p = 0.464	Na	0.09 (0.44), p = 0.829	Na	0.10 (0.25), p = 0.693
BMI (-)	0.14 (0.16), p = 0.364		0.04 (0.08), p = 0.630	Na	0.06 (0.14), p = 0.671	Na	0.002 (0.08), p = 0.979
%FM (%)	0.04 (0.70), p = 0.950	Na	**0.77 (0.34), p = 0.025**	Na	-0.01 (0.62), p = 0.982	Na	**0.93 (0.46), p = 0.045**
FM (kg)	0.05 (0.51), p = 0.924	Na	**0.53 (0.25), p = 0.034**	Na	0.24 (0.45), p = 0.594	Na	0.44 (0.26), p = 0.092
FFM (kg)	0.31 (0.39), p = 0.431	Na	-0.23 (0.20), p = 0.233	Na	-0.04 (0.35), p = 0.914	Na	-0.24 (0.20), p = 0.243
TBW (kg)	0.39 (0.40), p = 0.339	Na	-0.11 (0.20), p = 0.584	Na	0.12 (0.36), p = 0.742	Na	-0.15 (0.21), p = 0.480
TC (mg/dL)	-0.15 (6.31), p = 0.081	Na	1.16 (3.12), p = 0.710	Na	5.04 (5.61), p = 0.370	Na	-0.45 (3.25), p = 0.889
TGL (mg/dL)	0.30 (9.40), p = 0.975	Na	-0.52 (4.69), p = 0.912	Na	2.43 (8.39), p = 0.773	Na	-0.31 (4.86), p = 0.949
HDL (mg/dL)	-3.79 (3.10), p = 0.224	Na	1.49 (1.55), p = 0.339	Na	0.24 (2.79), p = 0.931	Na	1.61 (1.61), p = 0.318
LDL (mg/dL)	2.97 (5.85), p = 0.613	Na	-0.09 (2.89), p = 0.976	Na	3.77 (5.18), p = 0.468	Na	-1.84 (3.00), p = 0.540
BG (mg/dL)	-0.47 (2.78), p = 0.866	Na	0.44 (1.38), p = 0.749	Na	-1.08 (2.47), p = 0.663	Na	0.88 (1.44), p = 0.541

*IL1A* – G risk allele, IL6 (rs1800795) – C risk allele. Table cells show model coefficients, coefficient’s standard error in parentheses and raw p values—statistically significant when p < 0.05. DOM – dominant model: at least one risk allele at both *loci*; REC – recessive model: homozygotes for the risk allele at both *loci*; HOM-HET – homozygote-heterozygote model including four possible genotype combinations, where HOM1 is the homozygote for the risk allele and HOM2 is the homozygote for the major allele.

## DISCUSSION

Numerous studies have confirmed that systematic physical activity, in addition to diet, plays a key role in the regulation of body mass and body composition and, as a consequence, in the prevention of obesity [20]. Although mutual muscle-to-fat signaling factors have been identified, we are only in the early stages of understanding the complex pathways in which they participate. Thus, knowledge of the structure, function, and genetics of cytokines, such as IL-1α and IL-6, is essential for the development of a sensible intervention strategy for obesity prevention by enabling accurate prediction of individual training results involving weight loss and improved health [21, 22]. This study aimed to examine the impact of the four polymorphic sites located in the *IL1A* and *IL6* genes on the posttraining changes in the body mass and composition parameters, lipid profile, and glucose levels in Caucasian women taking part in a 12-week training program. To our knowledge, this is the first novel study to evaluate the association between genotypes, haplotypes, gene–gene interactions and physical outcomes. Therefore, our results cannot be directly compared to previous studies.

When tested individually, the statistical analyses showed two significant genotype × training interactions (for TBW and BMI). First, the carriers of the *IL1A* rs1800587 CC genotype exhibited a decrease in TBW in response to applied training. This finding suggests that this genotype may be unfavorable for achieving the desired training-induced TBW changes. This genotype may cause faster water loss in women and lead to disorders in the body’s homeostasis. Previously, this polymorphism was associated with the levels of IL-1α expression; specifically, the TT genotype significantly increased the transcriptional activity of *IL1A* with respect to the CC genotype. Thus, an increase of the mRNA and protein levels was observed in the plasma of carriers of the TT genotype [14]. Additionally, several studies have reported that chronic inflammatory diseases, including obesity, rheumatoid arthritis, Alzheimer’s disease, and periodontitis, are associated with the T allele [8, 23–25]. Thus, it was expected that the T allele carriers might show a higher BMI. However, Um et al. showed a reverse association between the T allele and a lower BMI value in obese healthy women [8]. Unfortunately, studies are lacking that describe the effect of the *IL1A* rs1800587 polymorphism on the posttraining changes in TBW and other body composition parameters, so further research is needed to confirm our observations.

Second, the carriers of the *IL6* rs1800797 GG and GA genotypes demonstrated a significant decrease in BMI in response to regular exercise. This result implies that these genotypes may be considered favorable factors for achieving the planned training-induced BMI change. Thus, *IL6* rs1800797 AA homozygotes should maintain more restrictive dietary habits and undergo more intense exercise than carriers of the G allele to achieve similar postworkout effects. The number of previous studies is small, and they have shown various results among different populations. Previously, the rs1800797 GG genotype was associated with higher levels of IL-6 expression. Some researchers have revealed the contribution of the *IL6* rs1800797 G allele to the development of type 2 diabetes and metabolic syndrome, suggesting a potential protective effect of the A allele against obesity and inflammation [26, 27]. On the other hand, Boeta-Lopez et al. did not report any significant connections between the rs1800797 G allele and obesity (categorized by BMI and waist circumference) and metabolic traits [28]. Genotyping in another study also showed a lack of significant association of rs1800797 with type 2 diabetes [29]. Our experiment only showed an association between GG and GA genotypes and a higher decrease in BMI in response to training, suggesting that these genotypes are favorable in the context of weight loss. This finding is inconsistent with some mentioned studies, which indicated that the G allele is the risk allele [26, 27]. However, interventional studies that include training programs are lacking, so we are unable to compare the results. More experimental studies are required to confirm the protective or harmful role of the *IL6* rs1800797 genotypes.

In addition, we identified a statistically significant association between the genotype and the glucose level; specifically, the carriers of the *IL6* rs1800795 GG and CG genotypes had an elevated glucose level during the entire study period compared with the CC genotype. Our results suggest that carrying these genotypes may have adverse effects on carbohydrate metabolism, and consequently, this polymorphism should be considered a genetic marker to determine predisposition to the development of hyperglycemia. This finding is in accordance with some previous studies, which found that the *IL6* rs1800795 G allele, generally associated with higher levels of IL-6 expression, is linked to a high risk of type 2 diabetes in many populations [26, 29, 30]. However, opposite results have been obtained in studies conducted on obese and type 2 diabetic participants, as well as some healthy individuals. In particular, the rs1800795 C allele has been associated with a high risk of obesity, cardiovascular disorders, and developing obesity-related metabolic disorders such as insulin resistance [31–33].

These inconsistent results related to individual SNP analysis led us to study the haplotypes of all *IL6* promoter variants and *IL6-IL1A* interactions. Only simultaneous analysis of multiple SNPs can provide additional unique information about the relationships between gene variants and observed phenotypic traits, as well as insight into the dependency among genetic markers [34], which was confirmed in this study. When the obtained results were included in the haplotype analysis performed for three polymorphic sites located in the *IL6* gene, no significant associations were revealed between the GGG, CGA, and GCG main haplotypes (rs1800795, rs1800796, and rs1800797, respectively) and physical outcomes. However, rare haplotypes such as GGA, CGG and CCG were linked to changes in several phenotypes, yet assessing individual haplotype effects was not possible. While these results did not better define the influence of the *IL6* promoter region on posttraining changes, they pave the way for future research. Previously, a few studies including haplotype analysis have revealed that the variability in the *IL6* gene is significantly associated with obesity, type 2 diabetes, and metabolic syndrome [33, 35]. Ramirez-Lopez et al. have shown significant associations between haplotype GCG/GCG (rs1800797, rs1800796, and rs1800795, respectively) and hyperglycemia; between GCG/ GCG and high high-sensitivity C-reactive protein; between AGC/AGC and obesity; and between GGG/GCG and low HDL levels [33]. In an obesity case-control study comparing haplotypes of the *IL6* promoter variants (rs1800797, rs1800796, and rs1800795, respectively), Hamid et al. found the AGC haplotype to be more frequent in the lean group than among obese participants, whereas the GGG haplotype was more frequent among obese subjects. The researchers also suggested that the rare haplotype AGC/GCG is associated with type 2 diabetes and that the common haplotype AGC/ GGG is associated with metabolic syndrome [35]. The mechanisms by which *IL6* promoter SNPs might cause an increased risk of obesity are still unknown, but it might be because of an effect on insulin resistance, suggesting a complicated role of IL-6 in body composition and glucose metabolism [36].

Previous results have revealed that IL-1α stimulates the expression of *IL6* gene transcripts and peptide production, which is regulated by cyclic adenosine monophosphate (cAMP) [37]. Thus, we performed an analysis of the interactions between the two genes encoding these interleukins, which showed that carrying TC-GG genotypes in both *IL1A* rs1800587 and *IL6* rs1800795 as well as *IL1A* rs1800587 and *IL6* rs1800796 may be associated with greater decreases in %FM and FM in response to applied training. This combination of genotypes may be considered beneficial, and carriers of these genotypes might more easily achieve the expected posttraining effects in terms of body composition changes. Additionally, the combination of the CC-CG genotypes in *IL1A* rs1800587 and *IL6* rs1800795 might be unfavorable for achieving the desired training-induced changes in lipidogram (TGL) parameters. In other study conducted on the Caucasian population, Maculewicz et al. (2022) analyzed the association between 5 polymorphisms of the interleukin 10 gene (*IL10*) and body composition parameters in 131 physically active participants. They revealed an association between BMI and CCGTA haplotype (rs1518111, rs1878672, rs3024496, rs3024498, and rs3024505, respectively) [38]. This finding indicates that there is a need to conduct additional studies involving other interleukin coding genes.

Although studies have confirmed that interleukin levels were determined according to weight change induced by lifestyle interventions [39], assessing individual variation in these marker levels is still very imprecise. Recently, it has been shown that people with the same genotype respond similarly to exercise and diet, indicating that some genetic variants play an important role in the determination of individual differences in response to lifestyle change [21, 22]. However, very few investigators have studied the biological variation of a particular analyte over a period of time, which makes it difficult to compare results. In addition, differences in the chosen methodology, inclusion criteria of participants, and interactions with the ethnic background and other genetic or environmental factors that influence the study population are reasons to explain why the search for genetic markers of the functional response of the human body to physical activities is very complicated and why the obtained results may be contradictory [22].

## CONCLUSIONS

The results of our experiment suggest that *IL1A* and *IL6* genotypes, either individually, in haplotype, or in gene-gene combination, may modify training-induced body mass, body composition, glucose level, and lipid profile changes in Caucasian females. The most important findings of our study are as follows: 1) carrying the *IL1A* rs1800597 CC genotype may be unfavorable for achieving the desired training-induced TBW changes; 2) carrying the *IL6* rs1800797 GG and GA genotypes may be favorable for achieving the planned training-induced BMI changes; 3) carrying the *IL6* rs1800795 GG and CG genotypes may have adverse effects on glucose metabolism; 4) only rare haplotypes (GGA, CGG and CCG) were linked to changes in several phenotypes, yet assessing individual haplotype effects was not possible; 5) carrying the TC-GG genotypes in both *IL1A* rs1800587 and *IL6* rs1800795 as well as *IL1A* rs1800587 and *IL6* rs1800796 may be associated with greater decreases in %FM and FM in response to applied training and may be considered beneficial; and 6) the combination of the CC-CG genotypes in *IL1A* rs1800587 and *IL6* rs1800795 may be unfavorable for achieving the desired training-induced changes in TGL levels.

## References

[1] Balkwill FR, Burke F. The Cytokine Network. Immunol Today. 1989; 10(9):299–304. 2686679 10.1016/0167-5699(89)90085-6

[2] Fain JN. Release of Interleukins and Other Inflammatory Cytokines by Human Adipose Tissue Is Enhanced in Obesity and Primarily Due to the Nonfat Cells. Vitam Horm. 2006; 74:443–77. 17027526 10.1016/S0083-6729(06)74018-3

[3] Lee MKS, Yvan-Charvet L, Masters SL, Murphy J. The Modern Interleukin-1 Superfamily: Divergent Roles in Obesity. Semin Immunol. 2016; 28(5):441–9. 27726910 10.1016/j.smim.2016.10.001

[4] Muoio DM, Leddy JJ, Horvath PJ, Awad AB, Pendergast DR. Effect of dietary fat on metabolic adjustments to maximal VO_2_ and endurance in runners. Med Sci Sports Exerc. 1994; 26(1):81–8. 8133743

[5] Pahlavani HA. Exercise Therapy for People With Sarcopenic Obesity: Myokines and Adipokines as Effective Actors. Front Endocrinol. 2022; 13:811751. 10.3389/fendo.2022.811751 PMC8892203 35250869

[6] Apte RN, Voronov E. Is Interleukin-1 a Good or Bad “guy” in Tumor Immunobiology and Immunotherapy? Immunol Rev. 2008; 222:222–41. 18364005 10.1111/j.1600-065X.2008.00615.x

[7] Di Renzo L, Bigioni M, Del Gobbo V, Premrov MG, Barbini U, Di Lorenzo N, De Lorenzo A. Interleukin-1 (IL-1) Receptor Antagonist Gene Polymorphism in Normal Weight Obese Syndrome: Relationship to Body Composition and IL-1 α and β Plasma Levels. Pharmacol Res. 2007; 55(2):131–8. 17174563 10.1016/j.phrs.2006.11.002

[8] Um JY, Rim HK, Kim SJ, Kim HL, Hong SH. Functional Polymorphism of IL-1 Alpha and Its Potential Role in Obesity in Humans and Mice. PLoS One. 2011; 6(12):e29524. 22216303 10.1371/journal.pone.0029524 PMC3246492

[9] Maculewicz E, Antkowiak B, Antkowiak O, Borecka A, Mastalerz A, Leońska-Duniec A, Humińska-Lisowska K, Michałowska--Sawczyn M, Garbacz A, Lorenz K, Szarska E, Dziuda Ł, Cywińska A, Cięszczyk P. The Interactions between Interleukin-1 Family Genes: IL1A, IL1B, IL1RN, and Obesity Parameters. BMC Genomics. 2022; 23(1):112. 35139823 10.1186/s12864-021-08258-x PMC8830010

[10] Goulart AC, Rexrode KM, Cheng S, Rose L, Buring JE, Ridker PM, Zee RYL. Association of Genetic Variants with the Metabolic Syndrome in 20,806 White Women: The Women’s Health Genome Study. Am Heart J. 2009; 158(2):257–262. 19619703 10.1016/j.ahj.2009.05.015 PMC2777704

[11] Benke KS, Carlson MC, Doan BQ, Walston JD, Xue QL, Reiner AP, Fried LP, Arking DE, Chakravarti A, Fallin MD. The Association of Genetic Variants in Interleukin-1 Genes with Cognition: Findings from the Cardiovascular Health Study. Exp Gerontol. 2011; 46(12):1010–9. 21968104 10.1016/j.exger.2011.09.005 PMC3689225

[12] Martinez-Nunez RT, Rupani H, Platé M, Niranjan M, Chambers RC, Howarth PH, Sanchez-Elsner T. Genome-Wide Posttranscriptional Dysregulation by MicroRNAs in Human Asthma as Revealed by Frac-Seq. J Immunol. 2018; 201(1):251–63. 29769273 10.4049/jimmunol.1701798 PMC6013048

[13] Ji H, Lu L, Huang J, Liu Y, Zhang B, Tang H, Sun D, Zhang Y, Shang H, Li Y, Lu H. IL1A Polymorphisms Is a Risk Factor for Colorectal Cancer in Chinese Han Population: A Case Control Study. BMC Cancer. 2019; 19(1):181. 30819119 10.1186/s12885-019-5395-9 PMC6394039

[14] Dominici R, Cattaneo M, Malferrari G, Archi D, Mariani C, Grimaldi L, Biunno I. Cloning and Functional Analysis of the Allelic Polymorphism in the Transcription Regulatory Region of Interleukin-1α. Immunogenetics. 2002; 54(2):82–6. 12037600 10.1007/s00251-002-0445-9

[15] Eder K, Baffy N, Falus A, Fulop AK. The Major Inflammatory Mediator Interleukin-6 and Obesity. Inflamm Res. 2009; 58(11):727–36. 19543691 10.1007/s00011-009-0060-4

[16] Vozarova B, Weyer C, Hanson K, Tataranni PA, Bogardus C, Pratley RE. Circulating Interleukin-6 in Relation to Adiposity, Insulin Action, and Insulin Secretion. Obes Res. 2001; 9(7):414–7. 11445664 10.1038/oby.2001.54

[17] Bastard JP, Jardel C, Bruckert E, Blondy P, Capeau J, Laville M, Vidal H, Hainque B. Elevated Levels of Interleukin 6 Are Reduced in Serum and Subcutaneous Adipose Tissue of Obese Women after Weight Loss. J Clin Endocrinol Metab. 2000; 85(9):3338–42. 10999830 10.1210/jcem.85.9.6839

[18] Akhter MS, Biswas A, Abdullah SM, Hobani Y, Ranjan R, Behari M, Saxena R. Influence of Interleukin-6 (IL-6) Promoter Gene Polymorphisms (−174G > C, −572G > C, and −597G > A) on IL-6 Plasma Levels and Their Impact in the Development of Acute Ischemic Stroke in Young Indians. Clin Appl Thromb Hemost. 2019; 25:1076029619854136. 31215220 10.1177/1076029619854136 PMC6714995

[19] Leońska-Duniec A, Jastrzębski Z, Jażdżewska A, Krzysztof F, Cięszczyk P. Leptin and Leptin Receptor Genes Are Associated With Obesity-Related Traits Changes in Response to Aerobic Training Program. J Strength Cond Res. 2018; 32(4):1036–44. 29373433 10.1519/JSC.0000000000002447

[20] Mayoral LPC, Andrade GM, Mayoral EPC, Huerta TH, Canseco SP, Canales RFJ, Cabrera-Fuentes HA, Cruz MM, Santiago ADP, Alpuche JJ, Zenteno E, Ruíz HM, Cruz RM, Jeronimo JH, Perez-Campos E. Obesity Subtypes, Related Biomarkers & Heterogeneity. Indian J Med Res. 2020; 151(1):11–21. 32134010 10.4103/ijmr.IJMR_1768_17 PMC7055173

[21] Switala K, Leonska-Duniec A. Physical Activity and Gene Association with Human Obesity. Balt J Health Phys Act. 2021; 13(4):99–111.

[22] Leońska-Duniec A, Ahmetov II, Zmijewski P. Genetic Variants Influencing Effectiveness of Exercise Training Programmes in Obesity – An Overview of Human Studies. Biol Sport. 2016; 33(3):207–14. 27601774 10.5604/20831862.1201052 PMC4993135

[23] Rainero I, Bo M, Ferrero M, Valfrè W, Vaula G, Pinessi L. Association between the Interleukin-1α Gene and Alzheimer’s Disease: A Meta-Analysis. Neurobiol Aging. 2004; 25(10):1293–8. 15465625 10.1016/j.neurobiolaging.2004.02.011

[24] Schulz S, Stein JM, Altermann W, Klapproth J, Zimmermann U, Reichert Y, Gläser C, Schaller HG, Reichert S. Single Nucleotide Polymorphisms in Interleukin-1gene Cluster and Subgingival Colonization with Aggregatibacter Actinomycetemcomitans in Patients with Aggressive Periodontitis. Hum Immunol. 2011; 72(10):940–6. 21672595 10.1016/j.humimm.2011.05.009

[25] Johnsen AK, Plenge RM, Butty V, Campbell C, Dieguez-Gonzalez R, Gomez-Reino JJ, Shadick N, Weinblatt M, Gonzalez A, Gregersen PK, Benoist C, Mathis D. A Broad Analysis of IL1 Polymorphism and Rheumatoid Arthritis. Arthritis Rheum. 2008; 58(7):1947–57. 18576312 10.1002/art.23592 PMC2533126

[26] Illig T, Bongardt F, Schöpfer A, Müller-Schulze S, Rathmann W, Koenig W, Thorand B, Vollmert C, Holle R, Kolb H, Herder C. Significant Association of the Interleukin-6 Gene Polymorphisms C-174G and A-598G with Type 2 Diabetes. J Clin Endocrinol Metab. 2004; 89(10):5053–8. 15472205 10.1210/jc.2004-0355

[27] Phillips CM, Goumidi L, Bertrais S, Ferguson JF, Field MR, Kelly ED, Mehegan J, Peloso GM, Cupples AL, Shen J, Ordovas JM, McManus R, Hercberg S, Portugal H, Lairon D, Planells R, Roche HM. Additive Effect of Polymorphisms in the IL-6, LTA, and TNF-α Genes and Plasma Fatty Acid Level Modulate Risk for the Metabolic Syndrome and Its Components. J Clin Endocrinol Metab. 2010; 95(3):1386–94. 20080841 10.1210/jc.2009-1081

[28] Boeta-Lopez K, Duran J, Elizondo D, Gonzales E, Rentfro A, Schwarzbach AE, Nair S. Association of Interleukin-6 Polymorphisms with Obesity or Metabolic Traits in Young Mexican-Americans. Obes Sci Pract. 2018; 4(1):85–96. 29479468 10.1002/osp4.138 PMC5818745

[29] Saxena M, Srivastava N, Banerjee M. Genetic Association of Adiponectin Gene Polymorphisms (+45T/G and +10211T/G) with Type 2 Diabetes in North Indians. Diabetes Metab Syndr. 2012; 6(2):65–9. 23153972 10.1016/j.dsx.2012.08.008

[30] Vozarova B, Fernández-Real JM, Knowler WC, Gallart L, Hanson RL, Gruber JD, Ricart W, Vendrell J, Richart C, Tataranni PA, Wolford JK. The Interleukin-6 (-174) G/C Promoter Polymorphism Is Associated with Type-2 Diabetes Mellitus in Native Americans and Caucasians. Hum Genet. 2003; 112(4):409–13. 12589429 10.1007/s00439-003-0912-x

[31] Stephens JW, Hurel SJ, Cooper JA, Acharya J, Miller GJ, Humphries SE. A Common Functional Variant in the Interleukin-6 Gene Is Associated with Increased Body Mass Index in Subjects with Type 2 Diabetes Mellitus. Mol Genet Metab. 2004; 82(2):180–6. 15172007 10.1016/j.ymgme.2004.04.001

[32] Goyenechea E, Parra D, Martínez JA. Impact of Interleukin 6-174G > C Polymorphism on Obesity-Related Metabolic Disorders in People with Excess in Body Weight. Metabolism. 2007; 56(12):1643–8. 17998015 10.1016/j.metabol.2007.07.005

[33] Ramírez-López G, Portilla-de Buen E, Sánchez-Corona J, Salmerón-Castro J, Mendoza-Carrera F. Interleukin-6 Polymorphisms Are Associated with Obesity and Hyperglycemia in Mexican Adolescents. Arch Med Res. 2013; 44(1):62–8. 23142265 10.1016/j.arcmed.2012.10.019

[34] Liu N, Zhang K, Zhao H. Haplotype-Association Analysis. Adv Genet. 2008; 60:335–405. 18358327 10.1016/S0065-2660(07)00414-2

[35] Hamid YH, Rose CS, Urhammer SA, Glümer C, Nolsøe R, Kristiansen OP, Mandrup-Poulsen T, Borch-Johnsen K, Jorgensen T, Hansen T, Pedersen O. Variations of the Interleukin-6 Promoter Are Associated with Features of the Metabolic Syndrome in Caucasian Danes. Diabetologia. 2005; 48(2):251–60. 15645209 10.1007/s00125-004-1623-0

[36] Wallenius V, Wallenius K, Ahrén B, Rudling M, Carlsten H, Dickson SL, Ohlsson C, Jansson JO. Interleukin-6-Deficient Mice Develop Mature-Onset Obesity. Nat Med. 2002; 8(1):75–9. 11786910 10.1038/nm0102-75

[37] Robson RL, Westwick J, Brown Z. Interleukin-1-Induced IL-8 and IL-6 Gene Expression and Production in Human Mesangial Cells Is Differentially Regulated by CAMP. Kidney Int. 1995; 48(6):1767–77. 8587236 10.1038/ki.1995.475

[38] Maculewicz E, Mastalerz A, Antkowiak B, et al. The effect of IL10 gene polymorphism on obesity parameters in highly physically active young men. Biol Sport. 2022; 39(4):1117–1125. 36247965 10.5114/biolsport.2023.118336 PMC9536378

[39] Wong E, Freiberg M, Tracy R, Kuller L. Epidemiology of Cytokines: The Women on the Move through Activity and Nutrition (WOMAN) Study. Am J Epidemiol. 2008; 168(4):443–53. 18579536 10.1093/aje/kwn132 PMC2727275

